# Surgical site infections following short-term radiotherapy and total mesorectal excision: results of a randomized study examining the role of gentamicin collagen implant in rectal cancer surgery

**DOI:** 10.1007/s10151-014-1193-1

**Published:** 2014-07-04

**Authors:** A. Rutkowski, L. Zając, L. Pietrzak, M. Bednarczyk, A. Byszek, J. Oledzki, T. Olesiński, M. Szpakowski, P. Saramak, M. Chwalinski

**Affiliations:** 1 Department of Oncological Gastroenterology, Maria Sklodowska-Curie Memorial Cancer Centre, W. K. Roentgena 5, 02-781 Warsaw, Poland; 2 Department of Radiotherapy, Maria Sklodowska-Curie Memorial Cancer Centre, Warsaw, Poland; 3 Department of Clinical Trials, Maria Sklodowska-Curie Memorial Cancer Centre, Warsaw, Poland; 4 Department of General, Vascular and Transplant Surgery, Medical University of Warsaw, Warsaw, Poland; 5 Department of Surgery, St. Elizabeth Hospital, Warsaw, Poland

**Keywords:** Surgical site infection, Gentamicin collagen implant, Rectal cancer, TME

## Abstract

**Background:**

Despite the findings of several randomized clinical studies, the role of gentamicin collagen implant (GCI) in rectal cancer surgery is unclear. Local pelvic application of GCI following preoperative radiotherapy and total mesorectal excision (TME) was evaluated to determine the risk of surgical site infections (SSI).

**Methods:**

In this single-center trial, 176 patients with rectal cancer after preoperative, short-term radiotherapy (5 × 5 Gy) were randomized either to the study group in which GCI was used or in the control group without GCI. Prior to surgery and intraoperatively five patients were excluded from the study. The remaining 171 patients were analyzed; 86 were in the study group and 85 in the control group.

**Results:**

There were no statistically significant differences in the overall rate of early postoperative complications between the study and control group: 25.6 and 34.1 % respectively; *p* = 0.245, relative risk (RR) 0.750 [95 % confidence interval (CI) 0.471–1.195]. The reoperation rate was similar in both groups: 12.8 versus 9.4 %; *p* = 0.628; RR 1.359; (95 % CI 0.575–3.212). The total rate of SSI and organ space SSI were 22.2 and 15.8 % without differences between the study and control group. In patients without anastomotic leakage, the risk of organ space SSI was significantly reduced in patients who received GCI: 2.6 versus 13.0 %; *p* = 0.018.

**Conclusions:**

Application of GCI in the pelvic cavity after short-term preoperative radiotherapy and TME may reduce the risk of organ space SSI but only in the absence of anastomotic leakage.

## Introduction

Experience from several randomized studies has suggested that local application of gentamicin collagen implant (GCI) may reduce the rate of surgical site infection (SSI) [1–5], whereas other multicenter, randomized studies have shown GCI had no effect [6, 7]. Only four studies focused on patients with rectal cancer [2–4, 6] and only in one did all patients receive preoperative short-term radiotherapy [4]. In most of these studies, the GCI was inserted into the wound above the abdominal fascia or into the sacral wound after abdominoperineal resection (APR) and complete closure of the pelvic peritoneal floor at the level with the remnants of the levators. Following total mesorectal excision (TME), an empty cavity and specific type of surgical wound appear in the pelvic area. Moreover, the risk of infective complications in patients who undergo preoperative radiotherapy is higher than in patients who have surgery alone [8]. In one randomized study conducted by our group on rectal cancer patients, the GCI was inserted into the pelvic cavity after TME but only 50.6 % of patients received preoperative radiotherapy [3]. There were fewer early postoperative complications in the GCI group (20.7 vs. 37.5 %; *p* = 0.044). Analysis of this study showed that patients in the study group undergoing radical resection had significantly better overall survival (OS) and disease-free survival (DFS) than those allocated to the control group, mainly due to reduction of the incidence of distant metastases. The reasons for this remain unknown. In another randomized study evaluating the effect of GCI in the prevention of perineal wound complications after APR, there were no statistically significant differences seen in rates of cancer recurrence between the treatment and control groups [6]. Due to conflicting results of these studies, it was decided to initiate a confirmatory randomized trial. The main objective of the study was the evaluation of the rate of local recurrence and distant recurrence in patients after R0 resection. The oncological outcomes will published after the completion of follow-up. The second objective was the assessment of risk of SSI (superficial, deep and organ space) and total risk of postoperative complications. The current evaluation presented an impact of the GCI on the risk of surgical site infections.

## Materials and methods

### Study design

The local ethics committee at the Centre of Oncology in Warsaw approved the study. Participation in the study was open to patients with resectable rectal cancer who were eligible for preoperative short-term radiotherapy and TME. Preoperative inclusion criteria were pathology confirmed adenocarcinoma of the rectum located up to 12 cm from the anal verge, age ≥18 years, World Health Organization (WHO) performance score 0–1, no distant metastases, cancer stage cT3-4, N0-2, or cT2 N1-2, preoperative short-term radiotherapy with 5 × 5 Gy, and adequate results of blood count: leukocytes ≥3.5 × 10^9^/L, neutrophils/granulocytes ≥1.5 × 10^9^/L and hemoglobin ≥9.0 g/dL. All patients signed written informed consent. The exclusion criteria were presence of distant metastases, other primary cancer, allergy to gentamicin or collagen, pregnancy and concomitant disorders such as ulcerative colitis or Crohn’s disease.

### Gentamicin collagen implant (GCI)

The gentamicin collagen implant (Garamycin^®^ Innocoll, Athlone, Co., Westmeath, Ireland) contained 130 mg of gentamicin. The maximal concentration of gentamicin in serum after application of one GCI estimated to be about 2 µg/mL. During the first 12 h after surgery, the level of gentamicin in the drainage exudates is approximately 80–700 times higher than in the serum [3, 9]. In addition, GCI is highly water-soluble and even short periods of immersion in saline before implantation may causes large loss of gentamicin content [10].

### Randomization

The patients were randomized to either the study group in which GCI was used or to the control group in which patients underwent operation without adjunctive use of GCI. Randomization was carried out after radiotherapy and before surgery by telephone to the independent trial office. Balanced randomization lists were used. No stratification was made.

### Preoperative irradiation

In all patients, a total dose of 25 Gy in five fractions over 5 days was given. Three dimensional planning was used. Patients were irradiated using 15 MV photon beams. The target volume included the rectum, the mesorectum, the lymph nodes along the iliac internal vessels, the presacral nodes and the nodes at the obturator foramens. The interval between the end of radiotherapy and surgery could not be longer than 6 days but in patients for whom there were contraindications to surgery soon after the radiotherapy completion, it was allowed to extend the interval to 6–8 weeks.

### Surgical treatment

Prior to surgery, patients underwent one day of dietary restrictions and bowel preparation. All patients routinely received antibiotic prophylaxis consisting of intravenous injections of metronidazole 500 mg and cefuroxime 1,500 mg three times a day. The first dose was given during premedication in the operating theater. The preferable duration of the prophylaxis was 24 h with an acceptable 3-day option. Low molecular weight heparin was used as thrombosis prophylaxis.

Tumors located in the lower and middle part of the rectum were resected by the use of the TME technique. Tumors located from 10 to 12 cm from the anal verge were removed by subtotal mesorectal excision, but in these cases, the mesorectum was transected at least 3 cm below the level of the lower tumor border. A lateral pelvic lymphadenectomy was not performed. At the early stage of the operation, high ligation of the inferior mesenteric artery or superior rectal artery was carried out. In most cases of anterior resection, the double stapler technique and end-to-end anastomosis were used. Colonic pouches or protective diverting stoma creation was left to the discretion of attending surgeon. Low anterior resection was defined as resection with anastomosis up to 6 cm from the anal verge. All patients had pelvic cavity drainage for the first 48 h after the operation. For very low lesions, the extralevator type of APR was carried out. In patients who were randomly assigned to receive GCI 2 implants were inserted in the space created after mesorectal resection. The implants were not wetted before implantation, and the abdominal cavity was not washed after GCI application. In the case of APR, the implants were inserted via the perineal wound before complete closure of the pelvic peritoneal floor.

### Follow-up evaluations, endpoints and definitions

All postoperative complications within 90 days after operation were recorded. Data concerning complications after discharge were collected during routine control visits 30 and 90 days after surgery. No data were gathered on late surgical and postradiation complications. The endpoint for the current analysis was the total rate of SSI which included superficial incisional infections and organ space infections according to the Centers for Disease Control (CDC) definitions [11] and other postoperative complications. Intra-abdominal infections were defined according to 5th Edition (2010) of Scottish Surveillance of Healthcare Associated Infection Programme (SSHAIP) Health Protection Scotland (HPS). In accordance with this, organ space SSI was diagnosed if one of the following criteria were met:

- Patient has organisms cultured from purulent material from intra-abdominal space obtained during a surgical operation or from drainage or needle aspiration and
- Patient has abscess or other evidence of intra-abdominal infection seen during a surgical operation or histopathologic examination or patient has at least two of the following signs, fever (>38 °C), nausea, vomiting, abdominal pain and radiographic evidence of infection, e.g., abnormal findings on ultrasound, computed tomography (CT) scan, magnetic resonance imaging (MRI) or abdominal X-ray.

Categories of complications were assessed using the Dindo classification [12]. In this study, organ space SSIs were classified as intra-abdominal or intrapelvic abscess and/or peritonitis with or without clinically diagnosed anastomotic leakage. The diagnosis of anastomotic leakage was based on digital rectal examination or observation of fecal material in the drain and confirmed radiologically in CT pelvic scan or by laparotomy. Intra-abdominal pelvic abscess near the anastomotic leakage site was considered a result of the leakage only when leakage was confirmed. Such a complication was classified as organ space SSI caused by anastomotic leakage. If anastomosis dehiscence was not confirmed, intra-abdominal abscess, peritonitis or purulent drainage from the pelvis were classified as organ space SSI without anastomotic leakage. This category of complications also included patients with pelvic pain and the fever above 38 °C lasting more than 48 h with suspected inflammatory infiltration of soft tissue in the pelvis on CT examination but without evidence of anastomotic leakage or abscess.

### Statistical analysis

All data were collected in case report forms. Standard methods of descriptive statistics were employed for demographic data. The calculation of the sample size was based on the primary endpoint of the study which was local recurrence and distant recurrence in patients after R0 resection. To this end, there should have been 176 randomized patients. As far as the study power for detecting differences in perineal wound complication rate, assuming there is a 30 % postoperative complication rate after preoperative radiotherapy and radical resection of rectal cancer, to detect 50 % reduction of complications at the significance level of 95 % and power of 80 % between two treatment-assigned groups, more than 200 patients would be needed. The analyses were carried out according to the intention-to-treat principle. Differences in proportions were assessed using the Chi-square test or Fisher’s exact test. Continuous variables were compared using the Mann–Whitney *U* test. For all tests, the statistical significance was accepted at *α* = 0.05. The data were analyzed with SPSS 14 for Windows (SPSS, Chicago, IL, USA).

## Results

**Fig. 1:**
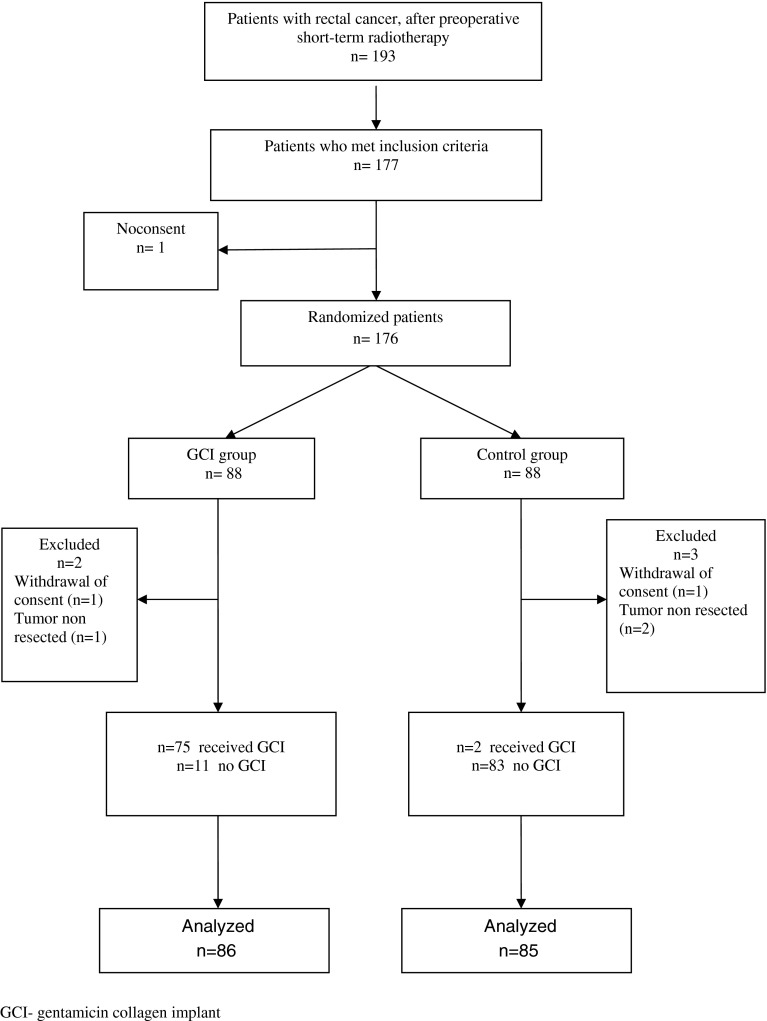
Profile of the study

From January 2008 to September 2011, out of 193 patients with rectal cancer who had undergone preoperative short-term radiotherapy, 177 (91.7 %) patients met all inclusion criteria. One patient did not agree to participate in the study. Altogether, 176 patients were randomized. However, immediately prior to surgery, two patients withdrew consent. Intraoperatively, another three patients were excluded, leaving 171 patients who were analyzed; 86 in the GCI group and 85 in the control group (Fig. 1).

### Patient characteristics

Patient characteristics were well balanced between the treatment assignment groups (Table 1). In total, 115 men (67.3 %) and 56 women (32.7 %) with a mean age of 63 years were analyzed. Median distance between the anal verge and lower tumor border was 5.3 cm. Surgery within one week after completion of short-term radiotherapy was performed in 145 patients (85 %). The remaining 26 patients (15 %) were operated on 6–8 weeks after radiotherapy. One hundred and thirty-three patients (77 %) underwent TME. In the remaining 40 patients (23 %) with high tumor, subtotal mesorectal excision was performed. A total of 50 (29 %) and 103 (60 %) patients underwent APR and anterior resection, respectively. A protective stoma was created in total of 18 % patients undergoing anterior resection: 15 % in the study group and 22 % in the control group (*p* = 0.476). The Hartmann procedure was performed in the remaining 18 patients (11 %). Median duration of surgical procedure was 145 min. In 151 patients (88 %), a 3-day course of antibiotic prophylaxis was used (78 in GCI group and 73 in control group).

**Table 1:** Patient characteristics

	GCI group *n* = 86	Control group *n* = 85	*p* value
Sex			0.550
Male	56	59	
Female	30	26	
Age			
Median (range)	63 (38–84)	63 (25–83)	0.789
WHO status			0.459
0	33	28	
1	53	57	
Median distance between the anal verge and lower tumor border in cm (range)	5.5 (0.5–12)	5.0 (0–12)	0.702
Type of surgery			0.621
Abdominoperineal resection	26	24	
Low anterior resection	34	36	
Anterior resection	19	14	
Hartmann procedure	7	11	
Protective stoma	8	11	0.476
J-pouch	1	0	1.000
End-to-end stapled anastomosis	512	48	1.000
Handsewn anastomosis		2	1.000
Mesorectum excision	64		0.496
Total (TME)	22	67	
Subtotal		18	
Surgery duration			0.394
<180 min	63	67	
≥180 min	23	18	
ypT			0.681
X	0	1	
0	3	1	
1	1	1	
2	28	21	
3	52	58	
4	2	3	
ypN			0.070
0	50	47	
1	28	20	
2	8	18	
ypM			0.368
0	82	84	
1	4	1	

*GCI* gentamicin collagen implant, *TME* total mesorectal excision, *WHO* World Health Organization

### Postoperative complications

Postoperative complications occurred in total of 51 (29.8 %) patients; 22 of 86 patients (25.6 %) in the GCI group and 29 of 85 (34.1 %) in the control group; *p* = 0.245, relative risk (RR) 0.750; (95 % CI 0.471–1.195). There were no 30-day postoperative deaths. Grade 3 or 4 complications according to the Dindo classification were observed in 20 patients (11.7 %): 11 of 86 patients (12.8 %) in the GCI group and 9 of 85 (10.6 %) in the control group; *p* = 0.813; RR 1.208; (95 % CI 0.528–2.766). In the remaining 31 patients, grade 1 or 2 complications occurred. Intraoperative complications occurred in 16 patients (6 patients in the study group and 10 in the control group). Application of GCI in such cases did not influence postoperative complications (*p* = 0.234). When surgery time exceeded ≥180 min, the rate of complications was 30.4 % in the GCI group and 50 % in the control group; *p* = 0.334; RR 0.609; (95 % CI 0.281–1.317). The reoperation rate was similar in both groups: 11 of 86 (12.8 %) versus 8 of 85 (9.4 %) respectively, *p* = 0.628; RR 1.359; (95 % CI 0.575–3.212). The total rate of clinical anastomotic leakage was 13.6 %; 17 % in the GCI group and 10 % in the control group, *p* = 0.392; RR 1.698; (95 % CI 0.611–4.722). A protective stoma had no effect on the observed differences in the rate of anastomotic leak between both groups. Reoperation was necessary in 11 patients (78.6 %) with anastomotic dehiscence; eight of nine (89 %) in the GCI group and three of five (60 %) in the control group, *p* = 0.505; RR 0.675; (95 % CI 0.318–1.432). In patients who underwent APR, abdominal and perineal wound infections occurred in 4 and 2 % respectively. Application of GCI in the pelvic cavity did not reduce the risk of infections.

### Surgical site infections (SSI)

The overall rate of SSI was 22.2 % (38 of 171 patients). Superficial or deep wound infection was diagnosed in 12 patients (7 %) and organ space SSI in 27 (15.8 %). Seven patients had both superficial and organ space SSI. If anastomotic leakage occurred, application of GCI did not affect the risk of organ space SSI. However, if there was no leakage, the risk of organ space SSI was significantly reduced in patients who received GCI: 2.6 % (95 %CI 0–6.2) versus 13.8 % (95 % CI 6.2–21.4); *p* = 0.018 (Table 2). Unplanned analysis of the subgroup of patients without anastomotic leakage showed that the type of surgery, TME technique and operative time had no impact on the risk of organ space SSI in both groups (Table 3). The median time of hospitalization was the same in both groups (median 8 days).

**Table 2:** Surgical site infection

	No. of SSI/total no. of patients (%)		*p* value	RR	95 % CI
	GCI group	Control group			
Superficial and/or deep incisional SSI	5/86 (5.8)	7/85 (8.2)	0.566	0.706	0.233–2.138
Organ space SSI	11/86 (12.8)	16/85 (18.8)	0.302	0.680	0.335–1.378
With anastomotic leakage^a^	9/53 (17)	5/50 (10)	0.389	1.698	0.602–4.722
Without anastomotic leakage^b^	2/77 (2.6)	11/80 (13.8)	0.018	0.189	0.043–0.825
Total SSI	16/86 (18.6)	22/85 (25.9)	0.275	0.719	0.407–1.271

*GCI* gentamicin collagen implant, *SSI* surgical site infection, *RR* relative risk, *CI* confidence interval

^a^Anterior resection only

^b^Patients with anastomotic leakage excluded

**Table 3:** Organ space surgical site infection without anastomotic leakage

	No. of organ space SSI/total no. of patients (%)		*p* value	RR	95 % CI
	GCI group *n* = 77^a^	Control group *n* = 80^a^			
Abdominoperineal resection	2/26 (7.7)	3/24 (12.5)	0.661	0.615	0.112–3.372
Low anterior resection	0/28 (–)	3/33 (9.4)	0.243	0.168	0.009–3.110
Anterior resection	0/16 (–)	0/12 (–)	(–)	(–)	(–)
Hartmann procedure	0/7 (–)	5/11 (45.6)	0.101	0.136	0.009–2.140
Total mesorectal excision (TME)	2/58 (3.4)	8/64 (12.5)	0.099	0.276	0.061–1.247
Intraoperative complications	0/6 (–)	3/10 (30.0)	0.250	0.225	0.014–3.720
Surgical procedure duration ≥180 min	1/20 (5.0)	5/18 (27.8)	0.083	0.180	0.023–1.399

*GCI* gentamicin collagen implant, *TME* total mesorectal excision, *RR* relative risk, *CI* confidence interval

^a^Patients with anastomotic leakage excluded

## Discussion

Earlier studies focused on the assessment of the risk of incisional and deep SSI depending on GCI application [1, 2, 4–7]. In most of these studies, the GCI was inserted into the wound above the abdominal fascia or into the sacral wound after APR. What is more, only four of the studies focused on patients with rectal cancer—Table 4 [2–4, 6]. In addition, the studies concerning the colorectal surgery occasionally show contradictory results. The surprising result of a randomized multicenter trial from the USA reveals a significantly higher rate of SSI in the GCI group [7]. The important bias of this study was the fact that the implants were soaked in saline prior to implantation. Lovering et al. [10] showed that after relatively short periods (2–60 s) of immersion of GCI in saline, losses of gentamicin were observed (from 6.7 to 40.5 %). This could have an impact on the efficacy of GCI. On the other hand, a review of clinical trials presented by de Bruin demonstrates that application of GCI can reduce the risk of SSI [13]. Brehant et al. [14] presented similar conclusions on the basis of a prospective analysis of 606 patients after colorectal surgery. The results of our previous study show positive effects of GCI, mainly in patients with surgery lasting longer than 180 min (19.2 vs. 40.8 %; *p* = 0.031) and in patients with intraoperative complications (10 vs. 57.9 %; *p* = 0.001) [3]. Contrary to other studies that focused on the assessment of the risk of incisional and deep SSI depending on GCI application, the current analysis also applies to organ space SSI. It is connected with the site of GCI application—the pelvic cavity. The results indicate that the application of GCI at the site of removed mesorectum reduces the risk of organ space SSI but only when the anastomotic integrity is maintained. Although in the current trial, GCI patients experienced a lower number of postoperative complications, the difference is not statistically significant and operating time did not influence the complication risk.

**Table 4:** Gentamicin collagen implant in rectal cancer surgery

Author	Material	% of preoperative radiotherapy	TME	Endpoint of the study	Results No. of complications/total no. of patients (%)		*p* value
					Study group	Control group	
Gruessner [2]	97 patients after APR	1	ND	Perineal wound infections	3/49 (6.1)	10/48 (20.8)	<0.05
Nowacki [3]	218 patients after APR, LAR, AR, Hartmann	53	Yes	Total postoperative complications	22/106 (20.8)	42/112 (37.5)	0.04
de Bruin [4]	40 patients after APR	100	Yes	Superficial and deep SSI	3/19 (18.8)	12/21 (57.1)	0.01
Collin [6]	102 patients after APR^a^	72.5	Yes	Perineal wound infections	19/52 (36.5)	21/50 (42)	0.57

*ND* not done, *APR* abdominoperineal resection, *LAR* low anterior resection, *AR* anterior resection, *SSI* surgical site infection, *AL* anastomotic leakage, *TME* total mesorectal excision

^a^18 patient with benign disease

The total rate of SSI and organ space SSI was 22.8 and 16 %, respectively. These results are similar to those reported by other authors [15–17]. The overall incidence of organ space SSI with and without anastomotic leakage was 9.3 and 8 %, respectively. Other authors have shown lower rates of organ space SSI with and without anastomotic leakage (2 and 0.8 %, respectively), but these results are for colon and rectal surgery [18]. There are many known risk factors for total and/or organ space SSI after elective colorectal surgery: tumor situated below peritoneal reflection (11 cm from the anal verge), types of operation (low anterior resection and Hartmann procedure), blood transfusion, poor general condition [American Society of Anesthesiologists (ASA) score 2 or 3], male gender, use of drainage, surgeon experience and stoma creation [15–19]. Most of these are also risk factors for anastomotic leakage [20–23]. Despite the fact that the difference in the rates of anastomotic leakage was not statistically significant, the high rate of clinical anastomotic leakage in the GCI group (18 %) may seem disturbing. Moreover, eight of nine (89 %) patients with anastomotic leakage who received GCI required reoperation, while in the control group, reoperation was necessary in only in two of four (50 %) patients with leakage. No connection between GCI application and anastomotic leakage has been shown in the literature [14, 24, 25]. There is also no data on the influence of high gentamicin concentrations and collagen upon the healing process of bowel anastomoses. There has been only one randomized study in which GCI was inserted around the colorectal anastomosis, but GCI had no effect on the risk of the leakage [3]. Although there is uncertainty as to whether organ space SSI in the form of a pelvic abscess at the site of the anastomosis is equivalent to leakage, the likelihood is that this is the case [19, 26]. Therefore, the distinction between organ space SSI with and without anastomotic leakage is justified. The results of the study showed that the risk of organ space SSI was significantly lower in those patients in whom GCI was used, but only when anastomotic leakage did not occur. This suggests that anastomotic leakage is such a significant risk factor for organ space SSI that the application of GCI does not prevent it. The low total rate of perineal wound infections (only 2 %) is surprising, while other authors have reported a higher percentage (>10 %) [2, 4, 6]. The reason for this phenomenon is unknown.

The current study has certain limitations. First, it should be stressed that assessment of postoperative complications was a secondary aim of the study, and therefore, the study lacks adequate power. Secondly, fewer patients received GCI due to protocol deviation. Thirdly, organ space SSI was not always confirmed by bacteriologic investigation. Bacteriological swabs were taken routinely during relaparotomy and from the wound if clinical symptoms of infection were present. The diagnosis of organ space SSI in the pelvic area without the presence of abscess and anastomotic leakage was based on clinical symptoms (pain and fever) and CT imagery (inflammatory infiltration of soft tissue of the pelvis) alone. Unfortunately, inflammatory lesions of soft tissue in the immediate postoperative period are virtually indistinguishable from early postoperative lesions not associated with infection. Therefore, the interpretation of CT imagery in connection with clinical symptoms depended in such cases entirely upon the surgeon and radiologist.

## Conclusions

Despite important limitations, the results of this study indicate that implantation of GCI in the pelvic cavity following short-term preoperative radiotherapy and TME for rectal cancer can reduce organ space SSI but only in the absence of anastomotic leakage.
